# Subtle genetic structure reveals restricted connectivity among populations of a coral reef fish inhabiting remote atolls

**DOI:** 10.1002/ece3.80

**Published:** 2012-03

**Authors:** Jim N Underwood, Michael J Travers, James P Gilmour

**Affiliations:** 1 Australian Institute of Marine Science, UWA Oceans Institute,Crawley, Western Austalia 6009; 2 Oceans Institute, University of Western Australia,Crawley, Western Austalia 6009

**Keywords:** *Chromis margaritifer*, damselfish, dispersal, north-west Australia, population connectivity, sweepstakes reproductive success

## Abstract

We utilized a spatial and temporal analyses of genetic structure, supplemented with ecological and oceanographic analysis, to assess patterns of population connectivity in a coral reef fish *Chromis margaritifer* among the unique and remote atolls in the eastern Indian Ocean. A subtle, but significant genetic discontinuity at 10 microsatellite DNA loci was detected between atoll systems corresponding with a low (≤ 1%) probability of advection across the hundreds of kilometers of open ocean that separates them. Thus, although genetic connections between systems are likely maintained by occasional long-distance dispersal of *C. margaritifer* larvae, ecological population connectivity at this spatial scale appears to be restricted. Further, within one of these atoll systems, significant spatial differentiation among samples was accompanied by a lack of temporal pairwise differentiation between recruit and adult samples, indicating that restrictions to connectivity also occur at a local scale (tens of kilometers). In contrast, a signal of panmixia was detected at the other atoll system studied. Lastly, greater relatedness and reduced genetic diversity within recruit samples was associated with relatively large differences among them, indicating the presence of sweepstakes reproduction whereby a small proportion of adults contributes to recruitment in the next generation. These results are congruent with earlier work on hard corals, suggesting that local production of larvae drives population replenishment in these atoll systems for a range of coral reef species.

## Introduction

A pelagic larval phase provides the opportunity for demersal marine organisms to disperse long distances by oceanic currents, and thus, contribute demographically and genetically to distant populations. However, how far and in what numbers larvae actually disperse away from their natal area and then survive to reproduce successfully depends on a wide range of interdependent biological and physical processes. Recent evidence suggests that such processes may restrict patterns of realized (or reproductive) population connectivity to smaller scales than those suggested by maximum dispersal potential (reviewed by Pineda et al. 2008). Since these patterns of larval connectivity underpin the maintenance, distribution, and diversity of marine populations, realistic estimates of the extent of larval supply and recruitment among populations are essential for predicting and mitigating the impacts of climate change and other human activities through the implementation of conservation strategies such as marine reserve networks (Jones et al. 2007; McCook et al. 2009). Although considerable progress in this field has been made recently (Cowen and Sponaugle 2009), practical difficulties in directly measuring larval connectivity mean that fundamental questions remain. In particular, we need to understand the spatial scales over which exogenous inputs of larvae supplement recruitment, how many individuals contribute to recruitment in the next generation, and how such connectivity patterns influence the temporal scales of recovery after disturbance.

Because genetic differences accumulate when populations are reproductively isolated and do not exchange genes, a spatial analysis of genetic variation provides an invaluable method for measuring patterns of population connectivity and yielding insights into the processes that drive them (Hedgecock et al. 2007). However, since the long-distance dispersal of a few individuals per generation is sufficient to homogenize genetic structure, but not replenish population numbers or increase short-term resilience to disturbance, the “tipping point” where populations become demographically independent usually occurs when the underlying genetic signal is weak (Waples and Gaggiotti 2006). Therefore, combining high-resolution genetic tools with other multidisciplinary approaches is crucial to elucidating ecological patterns of reproductive connectivity that are relevant to management, particularly in high gene flow species (Waples et al. 2008).

**Figure 1:**
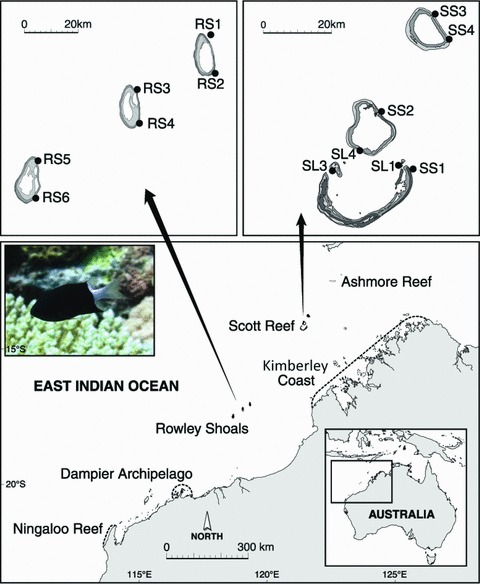
Map of the major coral reef systems in north-west Australia showing sampling sites of *Chromis margaritifer* (pictured in insert) collected from the atoll systems of Rowley Shoals and Scott Reef. Dashed lines represent well-developed coral reef habitat on the mainland, but note that *Chromis margaritifer* does not inhabit the mainland Kimberley coast or the Dampier Archipelago.

Coral atolls in the eastern Indian Ocean off north-west Australia have unique characteristics that make them not only high priorities for conservation, but also valuable study sites for investigating population connectivity. These remote coral atolls have a diverse array of flora and fauna (Bryce et al. 2009) and currently a relatively low intensity of most local human impacts. Additionally, there are only three well-developed emergent atoll systems along the edge of the continental shelf in this region. Each of these atoll systems are composed of three to four individual reefs, isolated from each other and the mainland by several hundreds of kilometers of open ocean (Fig. 1). Geographic isolation often means that pelagic marine larvae have a low probability of locating favorable habitat beyond their natal reef, and that locally derived recruits are likely to be crucial to population maintenance (see Strathmann et al. 2002). Consequently, compared with the mainland, offshore populations are often characterized by increasing genetic subdivision, endemism, and inbreeding, and reduced genetic diversity (e.g., Johnson et al. 1994; Frankham 1998; Bell 2008). The atolls of north-west Australia may therefore be particularly vulnerable to predicted changes in climate such as warmer waters and ocean acidification (Ayre and Hughes 2004), changes that in themselves exacerbate reproductive isolation by reducing population connectivity (Munday et al. 2009). From a methodological perspective, geographic isolation also means that signals of self-recruitment may be easier to detect than in more complex interconnected systems, particularly in organisms such as fish that have an extended pelagic larval phase with well-developed swimming and sensory behaviors.

There are two major surface currents that have the potential to transport larvae long distances among the atolls of north-west Australia; in the austral autumn and winter, a slow moving (∼0.2 ms^–1^) current originates near Indonesia and flows polewards along the continental shelf margin, while in the austral spring and summer, seasonal south-west winds induce a weak reversal of the current to the north-east (Cresswell et al. 1993; Holloway 1995). Satellite-tracked drifters suggest that transport of propagules between atoll systems would take between one and two months (Cresswell et al. 1993; Gilmour et al. 2009). These estimates are supported by studies of realized connectivity in hard corals. In situ observations following spawning, and spatial analyses of genetic structure of a brooding and broadcast spawning coral, suggest that the majority of recruits settle within a week and are retained close to their natal reef (Underwood et al. 2007, 2009; Gilmour et al. 2009).

In contrast to corals, most fish larvae spend at least several weeks in the plankton before they are competent to settle, and planktotrophic larvae can also delay metamorphosis (McCormick 1999). Although these larval characteristics may promote long-distance dispersal, fish larvae are clearly not passive particles; they can orientate themselves vertically in the water column, sustain swimming speeds that are faster than average current speeds, and can sense and swim toward reefs (Leis 2010). Considerable evidence is accumulating that these behaviors contribute to self-recruitment in many tropical (e.g., Doherty et al. 1995; Almany et al. 2007; Gerlach et al. 2007; Paris et al. 2007) and temperate fish species (e.g., Carreras–Carbonell et al. 2007; Galarza et al. 2009; Swearer and Shima 2011).

In addition to behavior, other more stochastic factors influence the spatial distribution and mixing of the larval pool in marine fish. In particular, research focusing on the genetic composition of recruits is yielding valuable insights into the “sweepstakes reproductive success” hypothesis (sensu Hedgecock 1994), in which variation in environmental conditions during fertilization, larval development, and settlement combined with high fecundity results in a relatively small proportion of individuals contributing to the majority of recruits in a particular generation (Selkoe et al. 2006; Buston et al. 2009; Christie et al. 2010; Villegas–Sanchez et al. 2010). This process greatly reduces effective population size relative to census size, and often leads to variation in numbers and genetic composition of recruits among local subpopulations (Turner et al. 2002; Hedrick 2005). In particular, telltale signals of sweepstakes reproduction include reduced allelic diversity, greater relatedness and heterozygote excess within recruit samples compared to adult samples, as well as temporal differentiation between recruit and adult samples (Hedgecock et al. 2007). Because reductions and spatial variations in breeding subpopulations may lead to reduced resilience, synthesizing location-specific data on reproductive output with changes in genetic composition among generations is important for informing spatial management strategies.

Here, we use interdisciplinary analyses to investigate for the first time the extent of reproductive connectivity among populations of a common damselfish (*Chromis margaritifer*) inhabiting the offshore atolls of north-west Australia. Our primary data are based on a spatial analysis of microsatellite DNA variation among samples of adults collected from the Rowley Shoals and Scott Reef (Fig. 1) to measure genetic connectivity across a hierarchy of spatial scales. We also compare genetic characteristics of recruits and adult samples to measure genetic changes between generations and test for the effects of sweepstakes reproductive success. Further, we analyze ecological and oceanographic data to aid interpretation of the genetic results. Specifically, we measure pelagic larval duration (PLD) of *C. margaritifer* to inform an oceanographic model estimating the probability for transport of passive particles between atoll systems in this region, as well as population densities at each atoll system, to test whether patterns of genetic structure are associated with potential reproductive output.

## Methods

### Study species

*Chromis margaritifer* is a highly abundant and widely distributed damselfish (Pomacentridae), occupying a variety of reef habitats, including inshore and offshore slopes and lagoons, from the central Pacific Ocean to the east Indian Ocean (Allen 1991). Like most damselfish, *C. margaritifer* is strongly site-attached, schooling within small home ranges (ca. <20 m^2^) following recruitment (pers. obs.). Therefore, migration among geographically separate populations is solely through dispersal of their planktonic larvae. Given that this species has a mean PLD of thirty or thirty-three days depending on location (Thresher et al. 1989; Wellington and Victor 1989), together with the sensory and swimming abilities of later stage fish larvae, the expectation is that connections within and between atoll systems will be stronger for this damselfish than for previously studied corals with more limited dispersal capacities (see Underwood et al. 2009). Counts of growth rings in otoliths from the largest adult specimens suggest that *C. margaritifer* is a short-lived (ca. one year) damselfish species (M. Travers, unpubl. data).

### Genetic sampling

In April 2008, *C. margaritifer* adults were collected by divers using a combination of hand nets, barrier nets, and clove oil from six sites at Rowley Shoals (*n*= 276) and seven sites at Scott Reef (*n*= 304; Fig. 1). On average, 45 fish were collected per site, and replicate sites were sampled on each reef (details in Table S1). In order to assess genetic characteristics of individuals capable of reproduction, adult samples comprised several cohorts of reproductively mature adults. Seven months later (October 2008), recently recruited fish (*n*= 98) were collected from three sites at Scott Reef (SL1, SL3, SS2) and one site at Rowley Shoals (RS3), with average samples sizes of 25 individuals per site (details in Table S2). Fish were categorized as recruits (15- to 30-mm tail length) and adults (>40-mm ail length; sensu Booth (2002)). Examination of growth rings from otoliths confirmed that this size class of recruits was all <3 months old (M. Travers, unpubl. data). A dorsal fin clip was placed in 100% ethanol (analytical grade) pending DNA extraction, and the rest of the whole fish was frozen for otolith extraction.

### Microsatellite genotyping

DNA for genotyping was extracted with the high-throughput membrane-based DNA extraction protocol of Ivanova et al. (2006) Quality and quantity of genomic DNA was ascertained through gel electrophoresis using 1% standard agarose (Amresco, Ohio, USA), and then diluted by one-third with millepore purified water (final concentration ca 10–20 ng) before PCR. The development of the microsatellite library, characterization of final 10 loci, and genotyping procedure were described in Underwood (2009). To mitigate and report scoring error of microsatellites, quality control procedures suggested by Bonin et al. (2004) and DeWoody et al. (2006) were implemented. Specifically, genotyping each individual involved the implementation of negative controls and the visual inspection of all automated allele calls, and individuals with suspect electropherograms were repeated. A genotype error rate (0.83%) was measured by repeating the genotyping procedure, from DNA extraction through to final allele scoring, using a subset of blind samples (*n*= 24) selected from three sites randomly spread across the sampling area.

Allelic patterns of the 580 adult fish collected from 13 sites, and 98 recruits collected from four sites, were calculated with GenAlEx v6.3 (Peakall and Smouse 2006). The number of alleles (*N*_A_), the unbiased expected heterozygosity (*H*_E_), the fixation index (*F*_IS_) at each of 10 microsatellite loci, and the number of private alleles at each site averaged across loci are presented in Tables S1 and S2. Tests for Hardy–Weinberg and linkage disequilibrium were conducted with FSTAT v2.9.3 (Goudet 1995) using the inbreeding coefficient *F*_IS_ and significance levels were based on 1000 permutations of alleles among individuals within sites and were adjusted with sequential Bonferroni correction for multiple tests when *P* < 0.05. Micro-Checker v2.2 (van Oosterhout et al. 2004) was used to detect and adjust for null alleles. Because, the vast majority of samples amplified across all loci (i.e., no obvious null homozygotes), we used Brookfield equation 1 to estimate null allele frequencies.

### Genetic analyses

To infer the strength of genetic connectivity across a hierarchy of spatial scales among the atolls of north-west Australia, we measured the amount of genetic variation that was geographically structured among adult samples with an analysis of molecular variance (AMOVA) framework in GenAlEx v6.3 (Peakall and Smouse 2006). We partitioned genetic variation between atoll systems (*F*_RT_), among sites relative to variation within each atoll systems (*F*_SR_), and among sites relative to overall variation (*F*_ST_). Additionally, we calculated the variation partitioned among sites at Rowley Shoals and Scott Reef system relative to the variation within that particular system (*F*_SR Rowleys_ and *F*_SR Scott_). To account not only for the high degree of variation within populations of microsatellite markers, but also for the effects that potential differences in effective population sizes might have on subdivision, we also calculated a standardized measure of all the *F*-statistics (*F′*_RT_, *F′*_SR_, and *F′*_ST_) according to the method of Meirmans (2006). To visualize the genetic relationships among adult samples, we performed a Principal Coordinates Analysis (PCoA; sensu Jombart et al. 2009) with pairwise *D*_S_ (Nei 1972) and *F*_ST_ estimates calculated in GenAlEx v6.3 (Peakall and Smouse 2006). *D*_S_ performed well in studies that evaluated the effectiveness of different genetic distances (Takezaki and Nei 1996; Paetkau et al. 1997), and provides a complimentary and independent comparison to the commonly used pairwise *F*_ST_. Pairwise matrices of *F*_ST_ (along with *F′*_ST_) and *D*_S_ estimates are given in Tables S3 and S4. We assessed the significance of spatial differentiation among allele frequencies of within each hierarchical grouping used in the AMOVA with a Fisher exact test implemented in Genepop v4.0 (Raymond and Rousset 1995). For this powerful test that is well suited to unbalanced samples sizes (Goudet et al. 1996), we used Markov chain parameters of 1000 iterations, 1000 batches, and dememorization number of 100.

To investigate further patterns of genetic connectivity and changes in genetic composition across generations, we assessed the genetic relationships among adults and recruit samples. Specifically, we tested for significant differentiation between recruit and adult samples at the four sites in which recruits and adults were collected (i.e., RS3, SL3, SL1, and SS2) with the exact tests implemented Genepop v4.0 (using the same Markov Chain parameters as spatial tests). Furthermore, to test explicitly for the effects sweepstakes reproduction, we calculated allelic richness (*R_S_*), the degree of relatedness within samples (*Rel*; Queller and Goodnight 1989), heterozygote deficiency (*F*_IS_:Weir and Cockerham 1984), and levels of geographic structure (*F*_ST_; Weir and Cockerham 1984), and tested for significant differences with randomization procedures (10,000 permutations) with FSTAT v2.9.3 (Goudet 1995). To visualize the temporal genetic relationships among recruit and adult samples relative to the amount of overall spatial variation, we conducted a PCoA in GenAlEx of pairwise *D*_S_ and *F*_ST_ estimates among all adult and recruit samples. Finally, because differences in levels of subdivision were detected among adult samples collected from the Rowley Shoals and Scott Reef systems (see Results) suggested differences in intensity of sweepstakes reproduction at each system, we tested also differences in allelic richness (*R*_S_) and heterozygote deficiency (*F*_IS_; Weir and Cockerham 1984) between adult samples from each atoll system with randomization procedures (10,000 permutations) as implemented in FSTAT v2.9.3 (Goudet 1995).

In addition to the above genetic analyses, we explored spatial patterns of genetic structure and propensity for self-recruitment of *C. margaritifer* with a number of methods. However, because these analyses yielded little additional information, we provide a brief description of methods and results in the Supporting Information.

### Otolith analysis

Because PLD has a pivotal influence on the *potential* for long-distance dispersal, varies among locations (see Bay et al. 2006), and has not been estimated in this region of north-west Australia for *C. margaritifer*, PLD was obtained from a subsample (*n*= 223) of fish collected for the genetic analyses from Rowley Shoals (*n*= 98) and Scott Reef (*n*= 125) to provide relevant dispersion times for the oceanographic modeling. Additionally, we tested whether differences in PLD were associated with differences in genetic structure (see results) at these two atoll systems. The sagittal otoliths were extracted and cleaned, and one from each fish was weighed, mounted on a glass slide, and ground following the method of Secor et al. (1991). The number of increments on each section was counted along the dorsoventral axis from the core to the edge and from the periphery to the core. We assumed that increments were deposited on a daily basis, and settlement marks corresponded to the type 1a abrupt settlement marks of Wilson and McCormick (1999). PLD was determined by counting the number of increments (days) between hatching (first increment) and settlement.

### Visual census surveys

Given that reproductive output is likely to influence the number of successful long-distance dispersers (see Steneck et al. 2009), we estimated density of *C. margaritifer* with underwater visual census to infer potential reproductive output and assess whether location-specific densities are associated with patterns of genetic structure. Permanent transects were established at 9-m depth and censused five times at Rowley Shoals (three locations), and 12 times at Scott Reef (six locations), between 1994 and 2008 (for details, see Heyward et al. 1995). Mean densities (±95% CI) across all sites and years were calculated for each atoll system.

### Oceanographic model

To explore the potential for advection of passive propagules by oceanic currents among the offshore systems of north-west Australia, we utilized the ConnIe model developed by CSIRO (Condie and Andrewartha 2008). This is a three-dimensional nonlinear hydrodynamic model that calculates circulation patterns forced by realistic wind, temperature, and salinity fields. A particle-tracking module was embedded within the model that allowed estimation of connectivity by tracking neutrally buoyant particles seeded randomly through the water column across the model domain. The circulation and particle movement calculations were conducted simultaneously, with particle positions updated every 10 min by the interpolated model current velocities. The probability that any two regions within the model domain were connected was computed for a range of dispersion times on a 0.1° geographical grid. We present the statistical outputs for six different scenarios: particles released from Rowley Shoals in the summer (first quarter) for twenty-eight and fifty-six days when the north-easterly flow was dominant; and then particles released from Scott Reef autumn (second quarter) over twenty-eight and fifty-six days when the south-westerly flow was the strongest. Additionally, since Ashmore Reef is the only other potential source population for Scott Reef and Rowley Shoals, we also present results of particles released from Ashmore Reef in autumn (during south-westerly flows) over twenty-eight and fifty-six days. Simulations of particles released from the mainland were not run because there are no known populations of *C. margaritifer* occurring along the adjacent mainland coast. We note that these simulations are for passive particles and do not incorporate larval behavior. However, given that larval behavior tends to increase local recruitment (Leis 2002; Cowen et al. 2006; Paris et al. 2007) and thus coarse oceanographic work probably overestimates the scale of ecological dispersal (Shanks 2009), this analysis provides estimates of upper limits for long-distance transport between atoll systems.

## Results

### General genetic characteristics

Genetic diversity of *C. margaritifer* populations was high, with an average of 18 alleles per locus per site and high proportions of expected heterozygotes (mean *H*_E_= 0.796) for the adults (details given in Tables S1 and S2). Consistent with the initial screening (Underwood 2009), significant heterozygote deficits were detected at 12 of the 13 sites in the adult samples and three of the four sites in the recruit samples, for each of the loci Cm_A115, Cm_B007, and Cm_D114. Analysis with Micro-Checker v2.2 indicated that these homozygote excesses were most likely due to null alleles at a frequency of between 0.09 and 0.17 per locus. This conclusion is supported not only by the presence of Hardy–Weinberg equilibrium at all other loci at all sites, but an absence of significant linkage disequilibrium. Thus, heterozygote deficits were unlikely to be caused by biological or sampling issues. Consequently, for all subsequent analyses, we present results based on an adjusted dataset calculated with Micro-Checker v2.2 to account for null alleles. However, compared with results of this adjusted dataset, patterns were highly congruent when only the seven loci that were in Hardy–Weinberg equilibrium were used.

### Spatial genetic structure of adults

The genetic structure of *C. margaritifer* indicated a lack of panmixis across the entire study, with low but significant genetic subdivision detected among all sites (*F*_ST_= 0.002, *P* < 0.001; Table 1). Importantly, all of this geographic structure in the genetic variation was entirely due to differences between systems (*F*_RT_= 0.002, *P* < 0.001) and not within systems (*F*_SR_= 0.000; Table 1). When *F*_ST_ estimates were standardized to within-population diversity (according to Meirmans 2006), levels of subdivision over the entire study area increased considerably, but the relative distribution of variation was the same; *F′*_RT_ and *F′*_ST_= 0.011, while *F′*_SR_ remained zero. Although variation due to differences among sites within systems was also zero when each atoll system was considered separately (*F*_SR Rowleys_ and *F*_SR Scott_= 0.000), variation among sites within Scott Reef increased when *F*_ST_ was standardized (*F′*_ST Scott_= 0.002), but remained zero at the Rowley Shoals. The exact test provided statistical support to these *F′* statistics, with significant differences detected among sites at Scott Reef (*P* < 0.001) but not at Rowley Shoals (*P*= 0.204).

**Table 1:** Results of the hierarchical analysis of molecular variance that partitioned genetic variation between atoll systems (*F*_RT_), among sites relative to variation within both atoll systems (*F*_SR_), and among sites relative to overall variation (*F*_ST_). Additionally, we calculated the variation partitioned among the sites relative to variation within each atoll system (*F*_SR Rowleys_ and *F*_SR Scott_). A standardized measure of all the *F* -statistics (*F′*_RT_, *F′*_SR_, and *F′*_ST_) was also calculated according to the method of Meirmans (2006). Exact tests were applied to asses statistical significance at each hierarchical level; ^*^ *P* < 0.001 and appears next to *F′* estimates.

Adults	Rowleys	Scott	All
*F*_RT_: between systems	–	–	0.002
*F*_SR_: within systems	–	–	0.000
*F*_ST_: among sites	0.000	0.000	0.002
*F′*_RT_: between systems	–	–	0.011^*^
*F*_SR_: within systems	–	–	0.000
*F′*_ST_: among all sites	0.000	0.002^*^	0.011^*^

**Figure 2:**
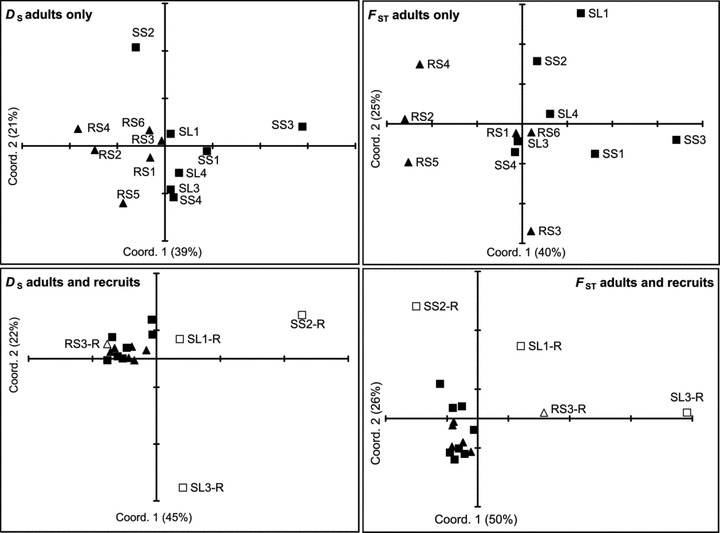
Principal Coordinate Analysis (PCoA) of genetic relationships of *Chromis margaritifer* among adult samples only (upper panels), and among adult and recruit samples (lower panels) from the atoll systems of Rowley Shoals and Scott Reef. Adult samples are indicated by full symbols, recruit samples by empty symbols, Rowley Shoals samples by triangles, and Scott Reef samples by squares. Estimates of pairwise genetic distances were derived from *D*_S_ (Nei's standard genetic distance) and *F*_ST_. The amount of variation explained by each axis is given in brackets.

The genetic discontinuity between samples of Rowley Shoals and Scott Reef adults detected by the AMOVA was illustrated in PCoA plots of Nei's standard genetic distance (*D*_S_; Fig. 2). Sites from the Rowley Shoals were positioned entirely on the left side plot in contrast to sites from Scott Reef, which predominantly occurred on the right side of the first axis. Although these patterns were not as obvious in the *F*_ST_ plot, sites from each system tended also to occur on either side of the first axis. Moreover, this PCoA also visually supported the greater local differentiation of genetic structure at Scott reef systems compared with the Rowley Shoals detected by the AMOVA and exact tests, with a much broader spread of sites from Scott Reef contrasting to the tighter clustering of sites from the Rowley Shoals. Finally, the differences in spatial subdivision of adult samples at each atoll system were associated with increased heterozygote deficiency at the Scott Reef system (*F*_IS Rowleys_= 0.086 and *F*_IS Scott_= 0.112; *P*= 0.009), but no differences in allelic richness (*R*_S Rowleys_= 16.15 and *R*_S Scott_= 15.83; *P*= 0182).

### Testing sweepstakes reproduction

Genetic characteristics of *C. margaritifer* recruits were generally consistent with expectations of sweepstakes reproduction (Hedgecock et al. 2007). Allelic diversity was significantly lower in samples of recruits compared with the adults (Table 2). Furthermore, geographic structure among sample and average relatedness within samples, were significantly greater for recruits than for adults (Table 2). The PCoA plots of *D*_S_ and *F*_ST_ that included recruit sites clearly illustrate the greater spatial genetic differentiation among recruit samples, which were broadly spread compared with the adult samples that formed a tight group (Fig. 2). Further, all samples of recruits were clearly separate from the adults on the *F*_ST_ plot, and a similar pattern was apparent (with the exception of RS3-R that clustered with adult samples) for the *D*_S_ plot. However, contrary to expectations of sweepstakes reproduction, recruit samples had a significantly greater proportion of heterozygote deficits (Table 2), while no significant pairwise differences were detected between adult samples and recruit samples collected seven months later with the exact tests (Table 3). Spatial differences were suggested between the Rowley Shoals recruit sample (RS3-R) and all three of the Scott Reef recruit samples (SL1-R, SL3-R, and SS2-R), but these differences did not remain significant after Bonferroni correction (Table 3).

**Table 2:** Results of analyses comparing allelic richness (*R*_S_), proportion of heterozygote deficits (*F*_IS_), relatedness (*Rel*), and *F*_ST_ between adult samples and recruit samples. Randomization procedures (10,000 permutations) implemented in FSTAT v2.9.3 (Goudet 1995) were used to test for statistical significant differences.

	Adults	Recruits	*P*-value
*R*_S_	10.01	9.64	0.031
*F*_IS_	0.100	0.139	0.008
*Rel*	0.000	0.008	0.001
*F*_ST_	0.000	0.005	0.001

**Table 3:** Results of the pairwise *F*_ST_ estimates (below diagonal) and *P* -values of exact tests (above diagonal) between recruit and adult samples. Main points discussed in the text are comparisons between samples of recruits and adults at each site (underlined), and comparisons between Rowley Shoals recruits and Scott Reef recruits (italicized). Significant values are given in bold, although the only comparison that remained significant after Bonferroni correction was between SL3-R and SL1-R.

	RS3	SL1	SL3	SS2	RS3-R	SL1-R	SL3-R	SS2-R
**RS3**	–	**0.008**	0.081	**0.024**	0.390	**0.012**	0.160	0.155
**SL1**	0.003	–	0.060	0.124	**0.013**	0.070	**0.008**	**0.025**
**SL3**	0.000	0.000	–	**0.007**	0.144	0.045	0.111	0.499
**SS2**	0.001	0.000	0.001	–	0.102	**0.028**	**0.006**	0.747
**RS3-R**	0.003	0.007	0.003	0.002	–	***0.003***	***0.005***	***0.042***
**SL1-R**	0.005	0.006	0.006	0.002	0.003	–	**0.001**	0.078
**SL3-R**	0.026	0.024	0.020	0.027	0.009	0.015	–	**0.004**
**SS2-R**	0.005	0.006	0.002	0.002	0.014	0.006	0.036	–

### PLD and population density

The mean (±95% CI) PLD of recently recruited *C. margaritifer* juveniles was thirty-five days (±8), and ranged from twenty-six to forty-two days (Table 4). There was little difference in PLD between the atoll systems, with a mean of thirty-four days (±8) calculated for fish from Scott Reef and thirty-six days (±12) for fish collected at the Rowley Shoals, and the same range at both reef systems (Table 4). The mean density (±95% CI) of *C. margaritifer* at the 9-m habitat was more than three times greater at the Rowley Shoals (116 fish 250 m^–2^± 7) than at Scott Reef (35 fish 250 m^–2^± 9).

**Table 4:** Summary of otolith analyses of *Chromis margaritifer* recruits collected from Rowley Shoals and Scott Reef.

	Rowley Shoals	Scott Reef
Mean (days)	36	34
Range (days)	16	16
Minimum (days)	26	26
Maximum (days)	42	42
Count (No. of fish)	98	125
Confidence Interval (days±95%)	12	8

### Oceanographic model

**Figure 3:**
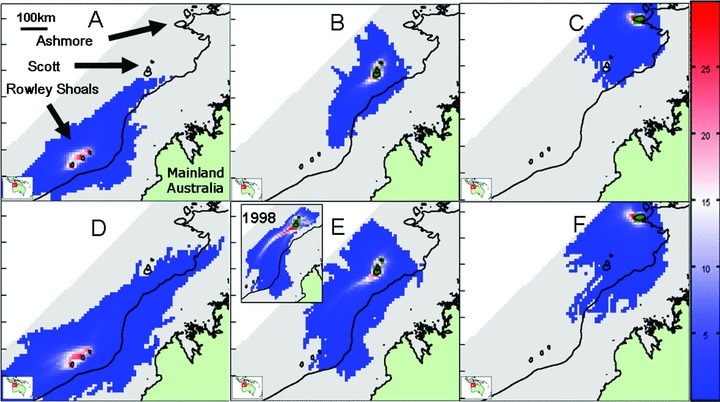
Probability distributions of passive particle transport between Rowley Shoals and Scott Reef estimated from a three-dimensional hydrodynamic model. Panels A, B, and C show results of particles run for twenty-eight days released from Rowley Shoals (in summer), Scott Reef, and Ashmore Reef (in Autumn), respectively. Panels D, E, and F show results of particles run for fifty-six days released from Rowley Shoals (in summer), Scott Reef, and Ashmore Reef (in Autumn), respectively. All results are based on particle distributions averaged across six years (1994–1999) apart from insert in panel E for 1998 only. The color bar indicates probability of connectivity, and green color is the release point of particles.

There was a clear reversal of oceanic currents from predominantly north-east in austral summer (illustrated in Fig. 3A and 3D) to south-west in the austral autumn (illustrated in Fig. 3B, 3C, 3E, and 3F) in all years. After one month of dispersal, irrespective of the season, the highest concentration of particles (10–30%) dispersed less than a few tens of kilometers of each atoll system, while the outer edge of the dispersal kernel (bounding the blue area of low probability at 0.1–1%) extended several hundred kilometers from the source reef (Fig. 3A, 3B, and 3C). For runs with a dispersion period of fifty-six days, the outer edge of the dispersal kernel of particles released from Rowley Shoals in summer reached the south section of Scott Reef (Fig. 3D), and similarly, particles released from Scott Reef in autumn reached the northern reef of Rowley Shoals (Fig. 3E). Further, the dispersal kernel of particles released from Ashmore Reef for fifty-six days completely encompassed Scott Reef but probability remained low (∼1%; Fig. 3F). Despite these low mean probabilities of dispersion between atoll systems, simulations over different years exhibited interannual variability. For example, in the second quarter of 1998, 5% of particles released from Scott Reef occurred within 30 kilometers of Rowley Shoals after fifty-six days (see Fig. 3E insert). The probability of dispersal between the reef systems during this year was much higher than the average across the six years in the same time period (main panel in Fig. 3E), and all other years individually (1994, 1996, 1997, 1999), in which outer edge of the dispersal kernel did not reach 100 kilometers from the Rowley Shoals (data not shown).

## Discussion

This study combined high-resolution genetic tools with other multidisciplinary approaches to reveal a pattern of locally restricted connectivity amidst a background of long-distance gene flow over multiple generations among the north-west Australian atolls in the damselfish *C. margaritifer*. These results not only augment a growing list of recent studies showing that locally produced larvae make important contributions to recruitment in many marine communities that include research on pomacentrids (e.g., Almany et al. 2007; Christie et al. 2010), marine fish more generally (e.g., Purcell et al. 2006; Gerlach et al. 2007; Knutsen et al. 2011), invertebrates (e.g., Johnson and Black 2006; van Oppen et al. 2008), oceanography (e.g., Cowen et al. 2006), and population modeling (e.g., Hastings and Botsford 2006), but also support the emerging consensus that localized recruitment and long-distance dispersal are clearly not mutually exclusive processes (Cowen et al. 2007; Jones et al. 2009; Planes et al. 2009; Puebla et al. 2009; Christie et al. 2010).

### Evolutionary connectivity between systems

Patterns of genetic structure of *C. margaritifer* populations at the Rowley Shoals and Scott Reef systems indicate sufficient connectivity to exchange most alleles that arise through mutation and replace other alleles that are lost through genetic drift over evolutionary time scales. High levels of genetic diversity were detected, and this diversity did not differ between systems. Moreover, a low level of spatial subdivision was detected across the scale of the study (*F*_ST_= 0.002 and *F′*_ST_= 0.011). The oceanographic model indicated that the potential for dispersal between the three major systems of the region (Rowley Shoals, Scott Reef, and Ashmore Reef) over a two-month period was low (≤ 1%) during most years, but that during one of the six years there was a 5% probability that propagules from Scott Reef were transported to within 30 kilometers of Rowley Shoals (Fig. 2F). Given that *C. margaritifer* reproduces throughout the year (J. Underwood and M. Travers unpubl. data) and is highly abundant at these offshore atolls, a large number of larvae are likely to be produced across a range of biophysical conditions, and when such oceanographic conditions combine with high reproductive output, occasional dispersal between the Rowley Shoals and the Scott Reef systems appears to be possible. Considering that potential source populations of this damselfish do not exist on the mainland, it therefore appears that a PLD of one to two months, the ability of larvae to locate suitable habitat, and physical connectivity via oceanic currents, has enabled larval exchange over multigenerational time scales to prevent major genetic divergence between these atoll systems.

Alternately, strong genetic connectivity over evolutionary time could be a product of past connections and equilibrium between migration, mutation, and drift has not yet been reached (see Benzie 1999). However, considering present-day coral reefs and oceanic currents were firmly established at least 10 Kya (Wyrwoll et al. 2009), an absence of physical “stepping stones” between the Rowley Shoals and Scott Reef systems, short generation times of *C. margaritifer* and high mutation rate of microsatellite markers, the genetic signal of potential historical connections is likely to have eroded over the thousands of generations since quaternary sea level and environmental changes (Waples and Gaggiotti 2006). Therefore, we conclude that contemporary (Holocene) influences are the dominant drivers of the overall genetic structure of *C. margaritifer* in north-west Australia.

### Ecological connectivity between systems

Amidst this background of low-level subdivision, we detected a significant genetic discontinuity between the Rowley Shoals and Scott Reef systems. Differentiation between the two atoll systems accounted for all of the geographic structure in the genetic variation of adult fish, and there was little overlap of Rowley Shoals and Scott Reef adult sites on PCoA plot (Fig. 2). Further, our census densities (35 fish 250 m^–2^ at Scott Reef and 116 fish 250 m^–2^ at Rowley Shoals) combined with estimates of suitable habitat area (ca 300 km^2^ at both systems) suggest that population sizes are in the tens of millions on these atolls. Thus, even considering that effective population size (*N*_e_) is likely to be several orders of magnitude smaller than census size (Turner et al. 2002), in an ecological context, a large *N*_e_ means that a migration rate (m) sufficient to maintain this levels differentiation detected here is far lower than the 10% that is probably necessary for demographically important exchange (see Hastings 1993; Waples 1998). Levels of genetic structure in this study are higher than for a damselfish *Stegastes partitus* in the Caribbean over similar spatial scales (Christie et al. 2010), and similar to that of the rock cod in the North Sea (Knutsen et al. 2011). Both these studies yielded direct evidence of self-recruitment through parentage tests and a capture-mark-recapture survey respectively, despite an absence of significant structure in the damselfish, and low levels of differentiation in rock cod. When placed in context of the geographic isolation of the atoll systems of north-west Australia and the sampling design of this study, together with the ecological and oceanographic results, we conclude that the genetic discontinuity between atoll systems detected here indicates that long-distance dispersal is unlikely to contribute to the ecological maintenance of *C. margaritifer* populations at the Rowley Shoals and Scott Reef systems on a generation-by-generation basis.

### Connectivity and sweepstakes reproduction within systems

In addition to an assessment of connectivity between atoll systems, we also gained important insights into population connectivity of *C. margaritifer* over scales of tens of kilometers within each system. The weak signal of geographic structure that was detected by the *F′*_ST_ within Scott Reef and not at the Rowley Shoals was confirmed by exact tests (Table 1), and visually illustrated by the adults only PCoA of pairwise *D*_S_ estimates (Fig. 2). Further, a significantly higher proportion of heterozygote deficits was detected at each adult sample at Scott Reef compared with the Rowley Shoals, suggesting that Scott Reef adult samples may well comprise admixed individuals that originate from genetically differentiated sources within the Scott Reef system. Additionally, although the recruit samples were separate from each other and from adult samples on the PCoA that included all samples (Fig. 2), when pairwise relationships between recruits and adults collected from the same site were considered, no significant differentiation was detected by the exact tests. Thus, this pattern of limited genetic variation through time despite considerable spatial variation within the Scott Reef system suggests that the genetic patchiness has a temporally stable component at least into the next generation, and that a degree of localized dispersal restricts reproductive connectivity within this atoll system.

The observed differences in genetic structure between Rowley Shoals and Scott Reef may be explained by the differences in population densities as well as the physical characteristics of the two systems. Assuming the same proportion of larvae will successfully disperse a particular distance between reefs, then reproductive output has the potential to influence greatly the number of recruits, particularly at the outer edge of a dispersal kernel (see Fig. 2 in Steneck et al. 2009). Our census estimates of density indicate that the reproductive output at Rowley Shoals is three times larger than at Scott Reef, and therefore may well explain the genetic signal of panmixis at the Rowley Shoals that was not observed at the Scott Reef system. Additionally, differences in physical characteristics of the Rowley Shoals and Scott Reef systems may also be contributing to differences in genetic structure of *C. margaritifer*. At Scott Reef, water flow is slowest inside the sheltered water of the semicircular-shaped southern reef and eddies formed by the eastern and western hooks of this reef complicate currents (J. Gilmour unpubl. data), and thus larval dispersal is likely to be restricted relative to the simple oval-shaped reefs of the Rowley Shoals (Fig. 1). Although deeper genetic structure was evident in a brooding and broadcast spawning coral inhabiting these atolls (Underwood et al. 2009), greater structure at Scott Reef compared with the Rowley Shoals was also detected in both of these hard coral species, suggesting that the physical structure of each atoll system has an important influence on patterns of larval dispersal and recruitment that is consistent among organisms with differing life histories. Increased sampling over multiple years together with fine-scale biophysical models is required to discriminate the relative influence of environmental and demographic parameters on the different patterns of connectivity observed within these atoll systems.

The genetic characteristics of *C. margaritifer* at Scott Reef provide an important and unique example of the effects of sweepstakes reproductive success in marine fish. Our most striking result was the greater differentiation (as measured by *F*_ST_, Table 2) among samples of recruits compared with adults detected with the randomization tests and illustrated by PCoA (Fig. 2). Additionally, allelic richness was lower and relatedness greater within recruits relative to the adult samples. However, we also observed some genetic characteristics that were contrary to expectations of sweepstakes recruitment, namely reduced heterozygosity in recruits and a lack of differentiation between recruit and adult samples. Although the precise causes of these unexpected patterns require further study, the lack of differentiation between generations suggests that a degree of locally restricted dispersal may well exist in conjunction with sweepstakes processes.

### Management ramifications

This study builds on previous work in north-west Australia on hard corals (Underwood et al. 2009) to provide a community perspective on connectivity (sensu Kinlan and Gaines 2003) for species with a wide variation of life histories that have implications for the resilience and management of these coral reef communities. Specifically, population connectivity in this benthic spawning damselfish, a brooding coral, and a broadcast spawning coral appears to be restricted between the atoll systems of Rowley Shoals and Scott Reef. This means that many communities inhabiting these geographically isolated atolls may well be demographically closed at the atoll system level or lower, and their recovery from disturbances will not be driven by input of new recruits produced from outside over time scales most relevant to management.

In addition to regionally specific inferences, this study also provides insights into patterns of population connectivity that are likely to be of benefit to the design of coral reef reserves in other systems. Strong connectivity among reefs in more widely dispersing species (e.g., abundant fish) inhabiting hydro-geographically uncomplicated systems is likely to buffer populations against severe and large-scale disturbance events, and in such cases, replicate protected areas at the reef scale (∼30 km) are likely to be adequate. In contrast, for species whose larvae recruit more locally (e.g., hard corals) or in systems where connectivity is restricted due to hydrodynamics (e.g., complicated archipelagos), protected areas may need to be replicated over smaller distances (∼10 km) if they are to maintain connections among them and thus enhance resilience to disturbances. If so, then a suitable size and spacing of protected areas for a range of species may reflect the lower end of dispersal distances (10–20 km). General rules of thumb are emerging that are congruent with these conclusions (Cowen et al. 2006; McCook et al. 2009; Roberts et al. 2010), but reconciling differences in connectivity among species and reefs and maximizing the resilience of entire communities to emerging disturbance regimes is a significant challenge to designers and managers of marine reserves.

## Supporting Information

**Table S1.** Details of the 10 microsatellite markers of*Chromis margaritifer* adults from Rowley Shoals and Scott Reef.

**Table S2.** Details of the 10 microsatellite markers from*Chromis margaritifer* recruits collected from Rowley Shoals and Scott Reef.

**Table S3.** Pairwise *F*_ST_ (below diagonal)*F′*_ST_ (above diagonal) estimates for*Chromis margaritifer* of adults samples collected from the Rowley Shoals and Scott Reef systems.

**Table S4.** Pairwise *F′*_ST_ estimatesfor *Chromis margaritifer* of adults and recruits collected from sites at Rowley Shoals and Scott Reef.

**Figure S1.** Spatial autocorrelation analyses of thegenetic correlation coefficient (*r*) as a function ofdistance for *Chromis margaritifer* at Scott Reef and Rowley Shoals in NWA calculated with GenAlEx v6.
