# Supplementary material for: Subtle genetic structure reveals restricted connectivity among populations of a coral reef fish inhabiting remote atolls
Source: Ecol Evol. 2012 Mar;2(3):666–79. doi: 10.1002/ece3.80 (PMC3399152; doi:10.1002/ece3.80)
Supplement: Supplementary file 1 [file ece30002-0666-SD1.doc]

Supporting Information

Table 1. Details of the ten microsatellite markers of *Chromis margaritifer* adults from Rowley Shoals and Scott Reef. Number of individuals genotyped at each site are given in brackets, along with the number of alleles (N_A_), the unbiased proportion of expected (H_E_) heterozygotes per locus and site, and F_IS_ calculated for each locus and each site (numbers in bold indicate significant heterozygote deficits). Also given are average number of alleles per locus (mean N_A_), average unbiased expected heterozygosity (mean H_E_), the average (mean F_IS_) for each loci across all sites, and the number of private alleles (P_VA_) at each site.

Table 2. Details of the ten microsatellite markers from *Chromis margaritifer* recruits collected from Rowley Shoals and Scott Reef. Number of individuals genotyped at each site are given in brackets, along with the number of alleles (N_A_), the unbiased proportion of expected (H_E_) heterozygotes per locus and site, and F_IS_ calculated for each locus and each site (numbers in bold indicate significant heterozygote deficits). Also given are average number of alleles per locus (mean N_A_), average unbiased expected heterozygosity (mean H_E_), the average (mean F_IS_) for each loci across all sites.

Table 3. Pairwise F_ST_ (below diagonal) F’_ST_ (above diagonal) estimates for *Chromis margaritifer* of adults samples collected from the Rowley Shoals and Scott Reef systems.

Table 4. Pairwise F’_ST_ estimates for *Chromis margaritifer* of adults and recruits collected from sites at Rowley Shoals and Scott Reef.

**Additional methods used to investigate self-recruitment and population structure**

First, we explored the propensity of damselfish larvae to self-recruit back to their natal reef within each atoll system by employing a spatial autocorrelation analysis to assess the extent of genetic affinity among geographically proximate fish (conducted in GenAlEx v6.3 Peakall & Smouse 2006). To test statistical significance of r at each distance class, the upper and lower bounds of the 95% confidence interval were defined by 1000 random permutations and if *r* was located within this confidence belt, the null hypothesis of no spatial genetic structure was accepted. Despite the large number of pairwise comparisons at each distance class (min *n* = 1227, max *n* = 14748), and the high power of the permutational test (Doub*le et a*l. 2005), the autocorrelation coefficient did not fall outside the 95% confidence belt at any distance class at either atoll system, providing evidence that genetic relatedness between individual damselfish was not due to the geographic distance within the atoll systems of Rowley Shoals and Scott Reef (Fig. 1).

Figure 1 Spatial autocorrelation analyses of the genetic correlation coefficient (r) as a function of distance for *Chromis margaritifer* at Scott Reef and Rowley Shoals in NWA calculated with GenAlEx v6. Dotted lines indicate the upper and lower bounds of the 95% confidence interval defined by 1000 random permutations.

Second, we explored utility of a parentage analysis (with the method of Christ*ie et a*l. 2010), but did not detect any parent/offspring pairs. This was not surprising, since applicability of parentage analysis relies heavily on the fraction of potential parents sampled, and therefore in our study system with large population sizes it is a practical challenge to sample more than a tiny fraction of parents (Saenz-Agudelo et al. 2009).

Third, we used the Bayesian programs STRUCTURE 2.3 (Pritcha*rd et a*l. 2000) to estimate the true number of populations without providing geographic information on collection location of each fish, and GENECLASS v2 (Pi*ry et a*l. 2004) to assign recruits to adult sites. As expected from the low levels of subdivision, there was not enough information in the data for STRUCTURE to identify more than one population, nor GENECLASS to confidently (<95% probability) assign recruits to populations (sites) sites of origin regardless of parameter selections.

References

Christie MR, Johnson DW, Stallings CD, Hixon MA (2010) Self-recruitment and sweepstakes reproduction amid extensive gene flow in a coral-reef fish. *Molecular Ecology*, **19**, 1042-1057.

Double MC, Peakall R, Beck NR, Cockburn A (2005) Dispersal, philopatry, and infidelity: dissecting local genetic structure in superb fairy-wrens (*Malurus cyaneus*). *Evolution*, **59**, 625-635.

Peakall R, Smouse PE (2006) GenAlEx 6: Genetic Analysis in Excel. Population genetic software for teaching and research. *Molecular Ecology Notes*, **6**, 288-295.

Piry S, Alapetite A, Cornuet JM, et al. (2004) GENECLASS 2: A software for Genetic Assignment and First-Generation Migrant Detection. *Journal of Heredity*, **95**, 536-539.

Pritchard JK, Stephens M, Donnelly P (2000) Inference of population structure using multilocus genotype data. *Genetics*, **155**, 945-959.

Saenz-Agudelo P, Jones GP, Thorrold SR, Planes S (2009) Estimating connectivity in marine populations: an empirical evaluation of assignment tests and parentage analysis under different gene flow scenarios. *Molecular Ecology*, **18**, 1765-1776.
